# Highly sensitive and specific Alu-based quantification of human cells among rodent cells

**DOI:** 10.1038/s41598-017-13402-3

**Published:** 2017-10-16

**Authors:** Kodai Funakoshi, Mozhdeh Bagheri, Ming Zhou, Ryoji Suzuki, Hiroshi Abe, Hideo Akashi

**Affiliations:** 1 Department of Microbiology and Immunology, Tohoku University Graduate School of Medicine, 2-1 Seiryo-machi, Aoba-ku, Sendai, 980-8575 Japan; 2 Department of Molecular Pathology, Tohoku University Graduate School of Medicine, 2-1 Seiryo-machi, Aoba-ku, Sendai, 980-8575 Japan; 3 Department of Anatomy, Akita University Graduate School of Medicine, 1-1-1 Hondo, Akita, 010-8543 Japan

## Abstract

Alu elements are primate-specific short interspersed elements (SINEs), over 1 million copies of which are present in the human genome; thus, Alu elements are useful targets for detecting human cells. However, previous Alu-based techniques for detecting human genomic DNA do not reach the theoretical limits of sensitivity and specificity. In this study, we developed a highly sensitive and specific Alu-based real-time PCR method for discriminating human cells from rodent cells, using a primer and probe set carefully designed to avoid possible cross-reactions with rodent genomes. From 100 ng of mixed human and rodent genomes, 1 fg of human genome, equivalent to 1 human cell in 100 million rodent cells, was detectable. Furthermore, *in vivo* mouse subrenal capsule xenotransplantation assays revealed that 10 human cells per mouse organ were detectable. In addition, after intravenous injection of human mesenchymal stem cells into NOD/SCID mice via tail vein, the biodistribution of human cells was trackable in the mouse lungs and kidneys for at least 1 week. Our findings indicate that our primer and probe set is applicable for the quantitative detection of tiny amounts of human cells, such as xenotransplanted human cancer or stem cells, in rodents.

## Introduction

The sensitive and specific quantification of human DNA using real-time quantitative PCR (qPCR) has played important roles in various fields, such as cancer diagnostics, cancer and stem cell research, and forensic science^1–3^. In cancer and stem cell research, the xenotransplantation of human cells into rodent systems is often used to evaluate the physiological functions of human cells. Over time within the host, most of the transplanted cells tend to be lost, but small populations survive and exhibit specific biological phenomena, such as tumor formation, cytokine release, or tissue repair^4,5^. In such experiments, it is necessary to ascertain the biodistribution or the number of transplanted cells in various organs. Thus, a highly sensitive and specific system is needed to detect rare populations of human cells among the rodent cells that vastly outnumber them.

Alu elements are primate-specific short interspersed elements (SINEs) about 300 nucleotides in length^6,7^. Alu elements are derived from the 7SL RNA gene, and a specific structure consisting of two 7SL RNA-derived monomers separated by an A-rich region formed after the evolutionary divergence of rodents and humans^8,9^. Alu elements are classified into three major subfamilies: Alu J (the oldest), Alu S (intermediate), and Alu Y (youngest)^7^. These major subfamilies have been further classified into dozens of branched subfamilies based on sequence similarity^10,11^. There are over 1 million Alu copies in the human genome, accounting for over 10% of the entire genome^12^. Because of their species specificity, small size, and extraordinarily high copy number, Alu elements are ideal targets for qPCR aimed at detecting human cells among the cells derived from other animal species^13–24^.

The minimum theoretical amount of human genomic DNA needed to detect Alu elements is 0.06–0.15 fg as calculated below, based on the standard genomic preparation method for PCR, in which the size of the genome fragments ranges from 2 × 10^4^ to 5 × 10^4^ base pairs (bp)^25^. As the average distance between each Alu element is 3 × 10^3^ bases^12^, most of the genome fragments would be expected to contain several Alu elements. Assuming that the genome within a human somatic cell is 6 × 10^9^ bp in length and 6 pg in weight^26^, then 1.2–3.0 × 10^5^ fragments with an average molecular weight of 0.02–0.05 fg can be generated. Given that the theoretical limit of detection in the qPCR system is as low as 3 copies^27^, Alu elements should be detectable in this amount of human genomic DNA.

On the other hand, there are several potential difficulties with Alu-based qPCR (Alu-qPCR), especially in the mixed background of rodent genomic DNA. First, in supraprimates, which include rodents, non-specific reactions could occur due to the presence of Alu-related 7SL RNA-derived SINEs, or B1 elements^8,9^. Second, a primer or probe might target multiple sites within an Alu sequence, because Alu elements consist of a direct repeat of two monomers with high sequence similarity^7^. Third, because of the extraordinarily high copy number of Alu elements and the presence of significant numbers of Alu pairs that are closely located^28^, the extension from one primer might reach the next Alu, and amplify the inter-Alu region^29^. Therefore, Alu-qPCR could unavoidably elicit non-specific reactions resulting in a high background signal. All of these possibilities could seriously affect the sensitivity and specificity of Alu-qPCR. In fact, the detection limits of Alu-qPCR reported so far in a mixed genomic background are on the pg scale^2^, whereas in Alu-qPCR for purified human genomic DNA, a detection limit as low as 10 fg has been reported^30^. To achieve a much higher sensitivity and specificity close to the theoretical detection limit, the above difficulties need to be overcome. However, to our knowledge, no previous study has adequately addressed these issues.

**Figure 1:**
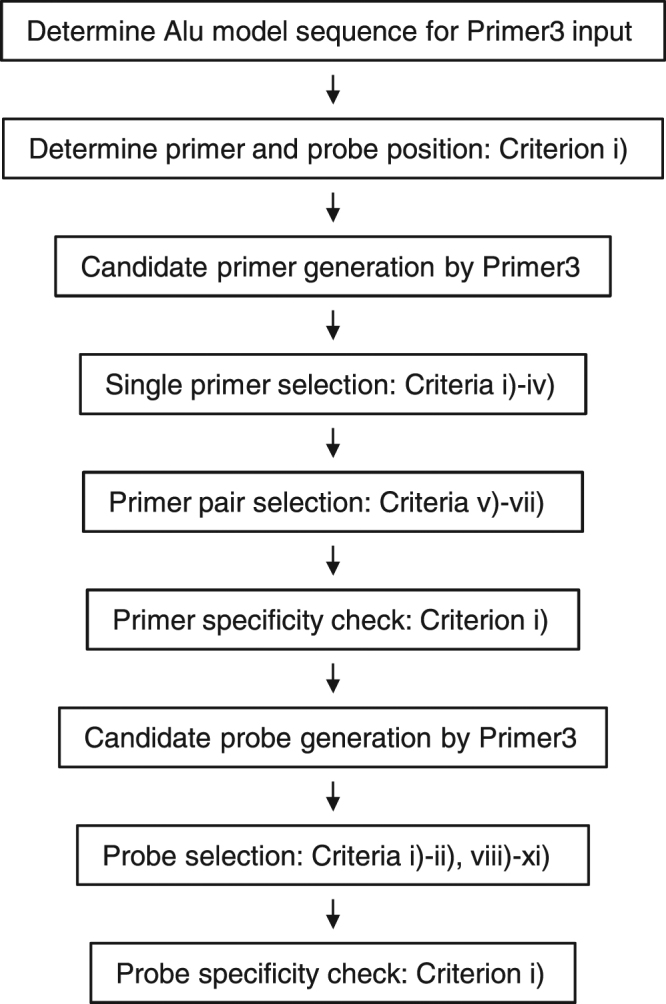
Overall flow of the procedure used to design Alu-specific primers and probes.

In the present study, we designed a new primers and probe set for Alu-qPCR with the aim of avoiding possible non-specific cross-reactions among the sequences of the primers, probe, and rodent genomes. For this purpose, we introduced several features into the design of the primers and probe. The overall flow of our design procedure is shown in Fig. 1. As a proof of principle, we aimed to demonstrate that our Alu-qPCR system was capable of discriminating 1 fg of human genomic material among 100 ng of the rodent genome, which is equivalent to 1 human cell among 100 million rodent cells. Furthermore, using an *in vivo* mouse model involving the xenotransplantation of human cells into the subrenal capsule, and also via tail vein, we showed that our Alu-qPCR system is of practical use for detecting human cells within a rodent system.

## Results

### Determination of the Alu model sequence

**Figure 2:**
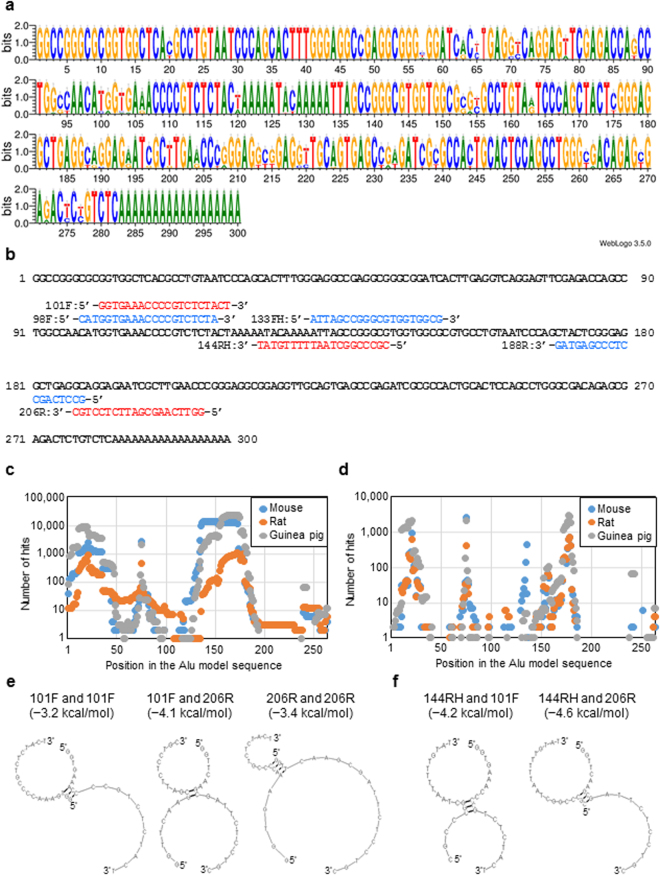
Design of Alu Primers and Probe. (**a**) Graphical representation of the Alu subfamily consensus sequences visualized by the WebLogo program. (**b**) The Alu model sequence and the primers and probe used in this study. The sequences of our primers and probe (101 F, 206 R, and 144RH, in red) and those of McBride *et al*. (98 F, 188 R, and 133FH, in blue) are depicted at the corresponding positions in the Alu model sequence (in bold). (**c**) The number of 19-nt sequences in the Alu model sequence that matched rodent genomic material. (**d**) The number of 19-nt sequences in the Alu model sequence showing a 100% match with rodent genomes. (**e**) Bimolecular secondary structures in homo- and heterodimers of the forward primer (101 F) and the reverse primer (206 R). (**f**) Bimolecular secondary structures between the primers and probe (144RH).

To design primers and a probe for Alu-qPCR, we first determined an appropriate Alu model sequence. The previously reported Alu consensus sequences do not represent a maximum consensus, because they were inferred from limited copies of Alu clones or Alu subfamily sequences^6,11,31^. Thus, to build an Alu model sequence for Alu as a whole, we referred to the recently developed Dfam database (version 1.3), which contains a comprehensive profile of Alu subfamily sequences^10^. The model consensus sequences from the 46 Alu subfamilies in the Dfam database were aligned using the Clustal W 2.1 program^32^, and then further aligned manually to determine the position of each nucleotide (Supplementary Fig. S1). The Alu subfamily sequences weighted by the number of members in each Alu subfamily were then visualized using the WebLogo 3.5.0 program (Fig. 2a)^33^. The graphical representation obtained using the WebLogo program was useful for clarifying the observed frequency of nucleotides at each position. For example, primers and probes that include position 57 in the Alu model sequence might not be appropriate for targeting a large number of Alu elements due to the low frequency of each nucleotide (Fig. 2a). Next, according to a position-specific frequency matrix (Supplementary Table S1), one Alu model sequence that represented the maximum consensus for the human Alu sequence was determined (Fig. 2b). We then used the Alu model sequence as a template for designing the Alu-specific primers and probes. The resulting Alu model sequence was closest to the consensus of Alu Sz among the Alu subfamilies, with a 2-nucleotide (2-nt) difference, and to that of Alu Sx, with a 3-nt difference, according to the Dfam search tool^34^, and differed by 14, 9, and 3 nt from the Alu model sequences reported by Deininger *et al*.^6^, Kariya *et al*.^31^, and Price *et al*.^11^, respectively (Supplementary Fig. S2).

### Primer and probe position

The homology of the Alu model sequence with the repetitive sequence that resides in rodent genomes was evaluated using the Censor program (http://www.girinst.org/censor/index.php)^35^. Almost all of the regions except for that between positions 117 and 135, which contained the Alu-specific A-rich sequence, were masked by the rodent PB1 SINE sequence that originated from the 7SL RNA gene and shares a common origin with human Alu elements^36^ (Supplementary Fig. S3). This finding suggested that we needed to choose the target sequences for specific primers and probes in the Alu model sequence very carefully.

To examine the homology of Alu subsequences to rodent genomes in detail, the Alu model sequence (positions 1–282) was homology-searched against rodent genomes using MegaBLAST. The MegaBLAST searches against the mouse, rat, and guinea pig genomes retrieved 23,099, 4,703, and 68,726 hits, respectively. To gain insight into where to design the specific Alu primers and probes, the number of hits that contained a 19-nt sequence starting from each position of the Alu model sequence was evaluated. However, the 19-nt subsequences from all of the positions in the Alu model sequence hit multiple sequences in the rodent genomes (Fig. 2c), indicating that the primers and probes would be selected from among sequences that were quite homologous to the rodent genomes. For simplicity, we decided to consider only 100%-identity hits, and not insertions, deletions, or mismatches of nucleotides that prevent contiguous matching. Of the above hits, 6,055, 2,951, and 13,298 showed 100% identity to contiguous Alu model sequences of 19 nt or longer (Fig. 2d). The results suggested that Alu subsequences of 20 nt that started from at least 144 positions in the Alu model sequence contained multiple 19-nt sequences that were 100% identical to rodent genomes (Supplementary Table S2).

As it was necessary to avoid sequences that were extremely homologous to rodent genomes when designing specific primers and probe for the Alu model sequence, we introduced the following constraints as design criteria: i) the primer or probe should not contain more than two 19-nt subsequences that perfectly match the genomic sequences of a specific rodent species, i.e. mouse, rat, or guinea pig; and ii) the primer or probe should be 20 nt or longer. These two criteria ensured that the primer or probe did not contain a 20-nt subsequence that perfectly matched any sequence in the rodent genomes.

### Single primer selection

Given that 18-nt contiguous matches between primers and rodent genomes were inevitable, it was necessary to control the specificities of primers and probes using the remaining mismatched nucleotides. When an 18-nt contiguous matched sequence lay within a 20-nt primer, 2 mismatches at either the 5′ or the 3′ end, or 1 mismatch at both the 5′ and 3′ ends, occurred. For mismatch discrimination, we considered that a strong (S) terminal mismatch (G or C) would lower the melting temperature (Tm) to a greater degree than a weak (W) mismatch (A or T)^37^, and that 3′-end mismatches would be refractory to PCR^38^. Although limiting the nucleotide to S at both ends and placing mismatches with rodent genomes at the 3′ end would maximize such mismatch-discriminating effects, these constraints were too strict to acquire a sufficient number of candidate primers. Therefore, the list of primers was narrowed down by adopting criterion iii) the first nucleotide from at least either the 5′ or the 3′ end was S (G or C), and if the first nucleotide from one end was not S, then the second nucleotide from the corresponding end was S.

According to criterion iii), at least 1 S would be introduced within 2 nucleotides from the 3′ end. It is reported that local stability of the 3′ end is important for PCR sensitivity, although instability at the 3′ end is also important for specificity^39^. Therefore, to avoid excessive stability at the 3′ end, we introduced an additional criterion iv) three Ss at the 3′ end were not allowed.

Using the Primer3web program version 4.0.0^40^, a list of 106 forward primers and 102 reverse primers was generated from the Alu model sequence. Of the forward primers, 52, 64, 68, and 93 primers fulfilled criteria i)-iv), respectively, and 13 fulfilled all of the criteria. Of the reverse primers, 51, 59, 69, and 79 primers fulfilled criteria i)-iv), respectively, and 14 fulfilled all of the criteria. After excluding primers whose positions did not allow them to be paired with any of the candidates, 11 forward primers and 8 reverse primers were selected.

### Primer pair selection

When high concentrations of primers and probes are used in PCR reactions, non-specific interactions among the primers and probes occur at local sites of low free energy. We assumed that unexpected interactions among the primers and probes would require a “stem” of a contiguous match as a scaffold. To select pairs of forward and reverse primers, the following criteria were introduced: any bimolecular structure of the homo- or heterodimer of the primers v) with a stem 3 nt or longer at the 3′ end, vi) with a stem 5 nt or longer, or vii) with a 4-nt stem that consisted of S only, was not allowed. Criteria v)-vii) were examined using the RNAstructure 5.6 program^41^. After excluding 3 forward and 3 reverse primers whose homodimers did not fulfill criteria v)-vii), hetero-dimers of the remaining 8 forward primers and 5 reverse primers were examined (Supplementary Table S3). The results revealed that, only one primer set with a forward (101 F; with a 5′ end starting from position 101 in the Alu model sequence) and a reverse (206 R; with a 5′ end starting from position 206 in the Alu model sequence) primer fulfilled all of the above criteria (Fig. 2b and e). The predicted Tm values of 101 F and 206 R were 58.7 °C and 59.3 °C, respectively.

### Primer specificity check

As the MegaBLAST search for the Alu model sequence did not retrieve all of the 100%-matched sequences, the candidate primers needed to be checked for specificity (Fig. 1). The specificity of the forward and reverse primers 101 F and 206 R was checked by a MegaBLAST search against rodent genomes (Table 1, left). The parameters for the MegaBLAST search were adjusted to retrieve all of the 100%-identity hits without mismatches. No perfect matches for the entire 20-nt sequences of our designed primers with rodent genomes were found. The one 19-nt hit of 101 F in the rat MegaBLAST search shown in Table 1 was considered to be human genomic contamination in the rat genome database for the following reasons: 1) The hit sequence was positioned within a 760-bp stretch of an isolated region in an unplaced genomic scaffold (positions 807–1566 in refseq ID: NW_007906637). 2) In NW_007906637, the sequence of the stretch between positions 1218 and 1496 was suggested to be the human Alu Y sequence by both Dfam (E-value = 2.9e−101) and Censor (Similarity = 0.9359). 3) In the BLAST search, the entire 760-bp region showed 98% identity to a sequence in human chromosome 12. For the 18-nt hits of 206 R in the mouse and guinea pig MegaBLAST search, the mismatch was located at the first 2 nucleotides, GG, from the 5′ end, and the predicted Tm was decreased to 55.0 °C, which was below the annealing temperature of 56.0 °C. Thus, the primer pairs 101 F and 206 R appeared to be specific for the human genome, with only marginal cross-reactions with rodent genomes.

**Table 1:** MegaBLAST search of the Alu primers and probe used in this study (left) and in the study by McBride *et al*. (right) against rodent genomes.

Mouse	20 nt	19 nt	18 nt		20 nt	19 nt	18 nt
101 F	0	0	0	98 F	0	1	0
206 R	0	0	1	188 R	—	7	20
144RH	0	0	1	133FH	64	283	862
**Rat**							
101 F	0	1*	0	98 F	0	4	4
206 R	0	0	0	188 R	—	23	41
144RH	0	0	0	133FH	0	0	2
**Guinea pig**							
101 F	0	0	0	98 F	0	1	0
206 R	0	0	1	188 R	—	91	316
144RH	0	0	1	133FH	1	36	91

The numbers of 100%-identity hits of 18–20 nt are shown. The lengths of 98 F and 188 R were 21 and 19 nt, respectively. The lengths of the other primers and the probe were 20 nt. *The 19-nt hit for 101 F against the rat genome might have been attributable to contamination by a human genomic sequence in the rat genome database, as described in the main text.

### Probe design

Because of the extremely high copy number of the Alu sequence in the human genome, one single primer can inherently amplify the inter-Alu genomic sequence in PCR reactions, which might result in the formation of amplified products with unpredictable and complex patterns^29^. To minimize the effects of such non-specific signals, we used hydrolysis probes.

In addition to criteria i) and ii), probe selection criteria corresponding to criteria v)-vii) for primer pair selection were introduced as follows: any bimolecular structure of the 101F-probe and 206R-probe, viii) with a stem 3 nt or longer at the 3′ end, ix) with a stem 5 nt or longer, or x) with a 4-nt stem that consisted only of S, was not allowed. The constraint for a probe-probe structure was not included in criteria viii)-x) because any probe to which a quencher is attached through a phosphate group at the 3′ end would not be a substrate for Taq polymerase. Since the G residue is reported to quench the fluorescent signal^42^, a probe-specific criterion was also introduced, criterion xi): the first base from the 5′ end should not be G.

Using the Alu model sequence between 101 F and 206 R as input (positions 121–186), 47 candidate probe sequences were generated by Primer3web. However, none of them fulfilled the above criteria. Therefore, we used a sequence complementary to the Alu model sequence as input for Primer3web, and another 47 sequences were retrieved. Of these candidate probes, 11 that started from position 144 or 145 and were 20–25 nt in length met the criteria. Among them, the 20-nt probe that showed the lowest free energy in any combination of bimolecular structural analyses was chosen (144RH; a reverse hydrolysis probe whose 5′ end starts from position 144 in the Alu model sequence, Fig. 2b and f). A MegaBLAST search revealed that 144RH did not contain a contiguous sequence longer than 18 nt in the rodent genomes (Table 1). The predicted Tm of 144RH was 57.8 °C, and the effect of the 2-nt mismatch in the mouse and guinea pig genomes on lowering the Tm was calculated to be −7.0 °C, suggesting that any cross-reaction of 144RH with rodent genomes would be marginal.

### Potential reactivity

Sequence analysis revealed that our set of primer pair (101 F and 206 R) and probe (144RH) perfectly matched corresponding parts of the model sequences of 9 Alu S subfamilies (Sx1, Sx, Sz, Sq2, Sg, Sq, Sx4, Sg4, Sq10) that covered 395,030 (40%) of the 996,723 total non-redundant Alu hits in the Dfam database^10^. This number might have been overestimated, because individual Alu element sequences may differ slightly from the model sequence of its Alu subfamily. When 101 F, 206 R, and 144RH were analyzed by a human MegaBLAST search (with default settings adjusted for short queries), 596,722, 576,583, and 516,661 hits were retrieved, respectively. Of these hits, the numbers that were mismatched by 0 to 2 nt at most are shown in Table 2. The number of perfectly matched hits shown in Table 2 might have been underestimated for actually detectable targets because, for example, the predicted Tm values of subsequences with a 1-nt mismatch at the 5′ end of 101 F, a 1-nt mismatch at the 5′ end of 206 R, and a 2-nt mismatch at the 3′ end of 144RH (56.1 °C, 59.0 °C, and 57.3 °C, respectively) were still above the annealing temperature (56.0 °C) used in our qPCR reactions.

**Table 2:** MegaBLAST search of our Alu primers and probe against the human genome.

	0 nt	1 nt	2 nt
101 F	88,454	272,877	402,087
206 R	72,063	247,776	428,782
144RH	9,839	68,235	131,708

The numbers of 20-nt 100%-identity hits (0 nt), and hits with a 1-nt (1 nt) and 2-nt (2 nt) mismatch at most are shown.

### Performance check

The sensitivity of the primers and probe was checked using a 10-fold serial dilution series of human genomic DNA ranging from 0.01 fg to 1 ng. For comparison, we also examined an already established and proven set of primers and probe^43,44^, originally developed by McBride *et al*.^13^. As it was possible to map this primer and probe set onto the Alu model sequence, we renamed them according to the position of their 5′ end in the Alu model sequence (forward primer 98 F, reverse primer 188 R, and probe 133FH in Fig. 2b). As shown in Fig. 2b, the primers and probe of McBride *et al*. were closely positioned to ours with partially overlapping nucleotides. The predicted Tm values for 98 F, 188 R, and 133FH were 59.5 °C, 61.5 °C, and 68.9 °C, respectively. Primer 98 F fulfilled our criteria ii), iv), vi), and vii); 188 R fulfilled criteria iii), iv), vi), and vii); and 133FH fulfilled our criteria ii), ix), and xi). Therefore, the primers and probe set of McBride *et al*. did not fulfill our criterion i), and perfectly matched many 19-nt sequences in the rodent genomes (Table 1, right).

**Figure 3:**
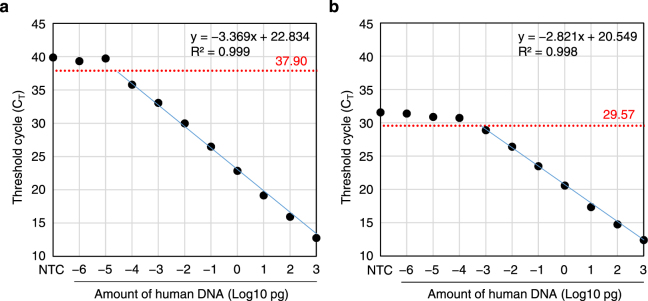
Standard Curve for Detecting Human Genomic DNA by qPCR Using the Primer and Probe Set Devised in this study (101 F, 206 R, and 144RH) (**a**) or that of McBride *et al*. (98 F, 188 R, and 133FH) (**b**). qPCR was performed for duplicate samples in one run, and the mean C_T_ values were plotted. Easy Dilution Buffer was used for the no-template controls (NTC). Threshold cycles of detection were set at 2 cycles below the C_T_ value for the NTC (red dotted lines), and the corresponding number of cycles is shown in red. The equations for linear approximations (blue lines) and R^2^ values are also shown.

We arbitrarily set the threshold cycle (C_T_) value at 2 cycles below that of the negative control as the threshold of detection (Fig. 3, red line), so that the C_T_ values of samples for negative controls or for a low amount of human genomic DNA outside the linear range were higher than this threshold value. The signals from the negative control sample may have resulted from a non-specific reaction among the primers and probe. Alu-qPCR using our primers and probe was able to detect as little as 0.1 fg of human DNA (Fig. 3a), corresponding to about 1∕60,000 of a human cell. The linear range of the standard curve extended over 7 orders of magnitude, with a correlation coefficient (R^2^) of 0.999 (Fig. 3a). Alu-qPCR with the primers and probe of McBride *et al*. detected 1 fg of human genomic DNA (Fig. 3b) The threshold of detection was 8.33 cycles lower than that using our primers and probe, and the linear range of the standard curve extended over 6 orders of magnitude (R^2^ = 0.998).

**Figure 4:**
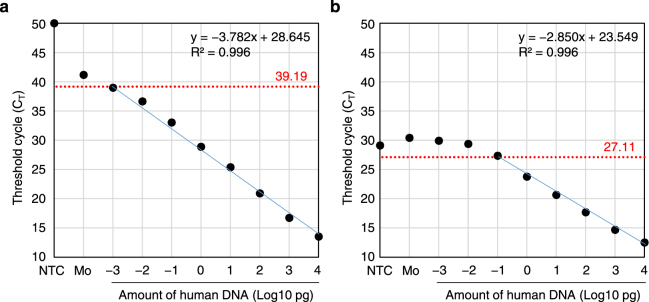
Standard Curve for Detecting Human Genomic DNA among a Total of 100 ng of Mixed Human and Mouse Genomic DNA Using the Primer and Probe Set Devised in this Study (101 F, 206 R, and 144RH) (**a**) and that of McBride *et al*. (98 F, 188 R, and 133FH) (**b**). qPCR was performed for duplicate samples in one run, and the mean C_T_ values were plotted. For non-detected samples, the C_T_ values were assumed to be for 50 cycles. Negative control samples included TE buffer for the no-template control (NTC) and a sample of mouse genomic DNA only (Mo). Threshold cycles of detection were set at 2 cycles below the lower C_T_ value between NTC and Mo (red dotted lines), and the corresponding number of cycles is shown in red. The equations for linear approximations (blue lines) and the R^2^ values are also shown.

Next, we evaluated the sensitivity and specificity of Alu-qPCR using mixed human and rodent genomes as templates. Various amounts of human genomic DNA were mixed with mouse genomic DNA to prepare 100 ng of total genomic DNA in a PCR reaction volume of 20 μl. The quantification limit of human genomic DNA by Alu-qPCR using our primers and probe was found to be as low as 1 fg, equivalent to 1 human cell among 100 million mouse cells (Fig. 4a). The threshold of detection of 39.19 cycles indicated a low level of non-specific signals. The linear range of the standard curve extended over 7 orders of magnitude with a correlation coefficient R^2^ of 0.996. Similar results were obtained in the presence of rat instead of mouse genome (Supplementary Fig. S4). On the other hand, Alu-qPCR with the primers and probe of McBride *et al*. detected 1 pg of human genomic DNA with a threshold of detection at 27.11 cycles (Fig. 4b), and the linear range of the standard curve extended over 4 orders of magnitude with a correlation coefficient (R^2^) of 0.996.

### Detection of human cells transplanted into mouse kidney

**Figure 5:**
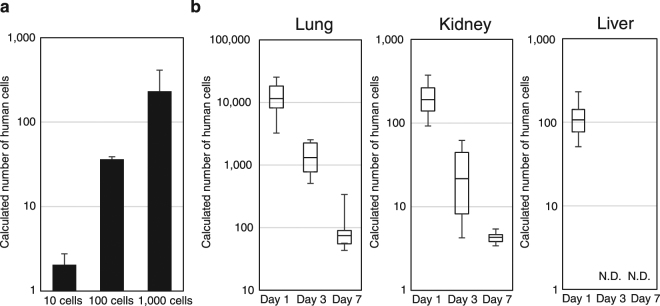
Detection of Human Cells in Mouse Xenotransplantation Models. (**a**) Detection of human cells in subrenal capsule assays. The indicated numbers of NHDF cells were injected into mouse subrenal capsules, and the numbers of cells calculated from the Alu-qPCR experiments are shown. qPCR was performed for triplicate samples in one run. Data represent the means, and bars indicate standard deviations for n = 3. (**b**) Detection of human cells in mouse organs at 1 day, 3 days, and 7 days after the injection of hMSCs into NOD/SCID mice via tail vein. qPCR was performed for triplicate samples in one run. The numbers of human cells calculated from the amount of human genomic DNA extracted from the lung, kidney, and liver are shown as box plots (n = 6). Whiskers represent minimum and maximum values, and the bold horizontal line represents the median. N.D. means not detected.

We further examined the validity of our Alu-qPCR system by xenotransplantation subrenal capsule assays in mice. The genomic DNA of whole mouse kidney was extracted 12 hours after the transplantation of 10, 100, or 1,000 human adult dermal fibroblasts (NHDF cells), and 100 ng of the genomic DNA was examined by qPCR. The number of human cells was then calculated from the qPCR calibration curve, the dilution factor of the samples, and the total amount of extracted genomic DNA. The Alu signal was reproducibly detected from the mouse kidney into which 10 NHDF cells had been transplanted (Fig. 5a). The somewhat smaller calculated number of cells compared with the actual number of transplanted cells was probably due to the loss of genomic DNA during our genome extraction procedure.

### Biodistribution of human mesenchymal stem cells after intravenous injection into SCID mice

Finally, we examined the biodistribution of human mesenchymal stem cells (hMSCs) using our Alu-qPCR system (Fig. 5b). We injected 0.5 million hMSCs into NOD/SCID mice intravenously via tail vein, and extracted genomic DNA from the mouse liver, lungs, and kidneys 1 day, 3 days, and 1 week after the injection. At 1 day (24 h) after injection, human genomic DNA was reliably detected from all of the organs, confirming the sensitivity of our Alu-qPCR system (Fig. 5b). In the lung, relatively abundant human genomic DNA (69,094.096 pg, or a median equivalent of 11,516 cells), possibly due to embolization of the lung vasculature^44^, was detected at day 1, and the amount was reduced to 446.840 pg (equivalent to 74 human cells) at 1 week. In the kidney, 1,147.022 pg of human genomic DNA (equivalent to 191 human cells) was detected at day 1, and the amount was still above the detection limit at 1 week (25.780 pg, or equivalent to 4 human cells). In the liver, 639.870 pg of human genomic DNA (equivalent to 119 human cells) was detected at day 1, and the number of cells declined thereafter and was undetectable on days 3 and 7.

## Discussion

In this study, we developed a highly sensitive and specific Alu-qPCR system for detecting human genomic DNA when mixed with rodent genomic DNA, placing emphasis on avoiding any non-specific binding among primers, probes, and rodent genomes. This was achieved through an original combination of 11 design criteria for the primers and probes intended to attenuate possible background signals from each source. We focused especially on avoiding 19-nt perfect matches with rodent genomes, and this goal formed the basis of criterion i). In terms of our criteria, we surveyed other Alu-qPCR systems that use hydrolysis probes, and found that at least two studies adopted criteria similar to our criterion i)^17,21^ (Table 3 and Supplementary Table S4). Although both of them targeted the Alu Y more specifically than the Alu S family to avoid amplifying genomes derived from non-human primates^17,21^, they still potentially targeted thousands of Alu members (Supplementary Table S4). Considering the theoretical detection limit of Alu-qPCR, we assume that their sensitivities would improve if some of our criteria were additionally applied. A further survey of Alu-qPCR using SYBR green chemistry revealed no other single primers that fulfill all of our criteria^14–16,18,20,22,23,30^ (Supplementary Table S5), indicating that our primers and probe were invented using a combination of design criteria distinct from all others.

**Table 3:** Conformity assessment of our design criteria to previously published primers and probes.

Primer sequence	Type	Criteria							Ref.
		i	ii	iii	iv	v	vi	vii	
CATGGTGAAACCCCGTCTCTA	F		X		X		X	X	^13^
GCCTCAGCCTCCCGAGTAG	R			X	X		X	X	^13^
GACCATCCCGGCTAAAACG	F	X		X	X	X	X		^17^
CGGGTTCACGCCATTCTC	R	X		X	X	X	X		^17^
GTCAGGAGATCGAGACCATCCT	F		X	X	X			X	^19^
AGTGGCGCAATCTCGGC	R	X		X		X			^19^
CTTGCAGTGAGCCGAGATT	F	X			X	X			^21^
GAGACGGAGTCTCGCTCTGTC	R	X	X	X	X				^21^
GAGGCGGGCGGATCA	F			X	X	X	X		^24^
CCCGGCTAATTTTTGTATTTTTAGTAG	R		X	X	X		X		^24^
GGTGAAACCCCGTCTCTACT	F	X	X	X	X	X	X	X	This study
GGTTCAAGCGATTCTCCTGC	R	X	X	X	X	X	X	X	This study
**Probe sequence**	**Type**	**Criteria**							**Ref**.
		**i**	**ii**	**viii**	**ix**	**x**	**xi**		
ATTAGCCGGGCGTGGTGGCG	H		X		X		X		^13^
CCCCGTCTCTACTAAA	H				X		X		^17^
AGCTACTCGGGAGGCTGAGGCAGGA	H		X		X	X	X		^19^
ACTGCAGTCCGCAGTCCGGCCT	H	X	X	X		X	X		^21^
CAGCCTGGCCAACATGGTGAAACC	H		X		X	X	X		^24^
CGCCCGGCTAATTTTTGTAT	H	X	X	X	X	X	X		This study

For sequences of 19 nt or less, we modified criterion i) to not allow 1 perfect match of full length or 2 perfect matches of 1-nt shorter subsequence with rodent genomes. F, R, and H: forward primer, reverse primer, and hydrolysis probe, respectively.

In designing a hydrolysis probe, it is often recommended that the probe Tm be 5–10 °C higher than that of the primers to enable the probe to bind to its targets earlier than the primers^45^ (Supplementary Table S4). However, this general guideline might not be appropriate for Alu-qPCR, as discussed below. The design of the probe 133FH followed the above guideline, because the Tm was 9.5 °C and 7.4 °C higher than those of the primers 98 F and 188 R, respectively. The high Tm of 133FH allowed it to bind to targets at the annealing/extension temperature (60.0 °C), even in the presence of several mismatches. For example, the predicted Tm of the 15-nt sequence from the 3′ end of 133FH (64.5 °C) was still above those of the primers and the annealing/extension temperature. As Taq polymerase can degrade the hydrolysis probe as long as the probe forms a stable duplex with a target^46^, then in addition to the 64 sites of 20-nt perfect matches with the mouse genome (Table 1), thousands of perfectly matched 15-nt sites would also be potential targets of 133FH (data not shown). This would significantly increase the fluorescent background signal in Alu-qPCR, where non-specific amplifications are hard to avoid. In fact, while the Alu-qPCR with 133FH performed well in samples containing only human genomic DNA, detecting quantities as small as 1 fg (Fig. 3b), the sensitivity dropped to 1 pg when the human genomic DNA was mixed with rodent genomic material (Fig. 4b). In contrast, the Tm of our probe 144RH (57.8 °C) was slightly lower than that of the primers (58.7 °C and 59.3 °C). Considering that the binding was completed within several seconds^47^, we assumed that the slightly lower Tm of 144RH would not have a serious effect on the results. In the presence of a 1-nt mismatch at the 5′ end and contiguous 3-nt mismatches from the 3′ end, the predicted Tm of 144RH (54.6 °C and 55.1 °C, respectively) was below the annealing temperature (56.0 °C). Thus, the only potential target site in the mouse genome was one 18-nt site (Table 1). Together, these findings suggest that one of the reasons for the low background signal threshold of our Alu-qPCR was the high specificity of the 144RH probe (Figs 3a and 4a).

There are several factors that might have caused fluctuations in the sensitivity of our Alu-qPCR system. 1) The fluorescent background signal of the negative controls used to determine the threshold of detection was derived from an unpredictable non-specific reaction among primers and probes and the rodent genomic DNA. In fact, the C_T_ values of the negative controls fluctuated somewhat between 40–45 cycles throughout the experiments, and the quantification limit for human genomic DNA among rodent genomic material fluctuated between 1 and 10 fg about once in every 4 experiments (data not shown). In these cases, the C_T_ values for 1 fg were still at least 1 cycle below those of the negative controls. 2) The amounts of Alu elements that were detectable by our Alu-qPCR system might have been prone to stochastic effects after extensive dilution. The sensitivity was determined by the number of Alu elements targeted by 144RH, which had the lowest number of target sequences among our primers and probe (Table 2). As the number of 144RH targets was roughly estimated to be 1–10% of the total Alu elements (Table 2), 1 targetable Alu element would be present in 0.03–0.3 fg of human genomic DNA, and thus close to the quantification limit of our Alu-qPCR system, possibly resulting in some stochastic effects.

Finally, our *in vivo* xenotransplantation experiments revealed that our Alu-qPCR system reliably detected human genomic DNA in mouse lung and kidney even at 1 week after the intravenous injection of human cells via tail vein (Fig. 5b), thus confirming that the system would be especially useful for cancer and stem cell research. Furthermore, the criteria adopted for our Alu-qPCR system might be applicable to the design of any primers and probes for studies requiring sensitive and specific detection. We believe that our set of primers and probe, and the approach we used for their design, could contribute to a variety of life science studies aimed at the sensitive and specific detection of human genomic material.

## Methods

### Cells and culture conditions

Human adult dermal fibroblast NHDF cells (CC-2511; Lonza, Basel, Switzerland) and human mesenchymal stem cells (hMSCs) (PT-2501; Lonza) were grown and maintained in Dulbecco’s modified Eagle medium (DMEM, low glucose; 10567-014, Thermo Fisher Scientific, Waltham, USA) containing 10% fetal bovine serum (FBS) and 0.1 mg/ml kanamycin sulfate, at 37 °C in an atmosphere of 5% CO_2_.

### Mice

Retired breeder C57BL/6 mice (C57BL/6JJmsSlc; Japan SLC, Hamamatsu, Japan) and six-week-old male non-obese diabetic/severe combined immunodeficiency (NOD/SCID) mice (NOD.CB17-Prkdc^scid^/J; Japan Charles River, Yokohama, Japan) were grown under specific pathogen-free conditions at the Center for Laboratory Animal Research, Tohoku University Graduate School of Medicine. Mice were assigned randomly to control and treatment groups, and no blinding was done. All control and treated mice were used for the analyses. The sample size for animal experiments was chosen based on our experience and published evidence from other groups. All animal experiments were conducted in accordance with the Tohoku University guidelines for animal experimentation. All of the experimental protocols were approved by the Tohoku University Committee for Safety Management of Animals.

### Oligonucleotides

Primers and probes were synthesized by FASMAC (Atsugi, Japan). The sequences of our primers were as follows: forward primer (101 F), 5′-GGTGAAACCCCGTCTCTACT-3′; reverse primer (206 R), 5′-GGTTCAAGCGATTCTCCTGC-3′. The sequence of our hydrolysis probe (144RH) was 5′-CGCCCGGCTAATTTTTGTAT-3′. The probe 144RH was labeled with the fluorescent reporter 6-carboxy-fluorescein (6-FAM) at the 5′ end and the fluorescent quencher Black Hole Quencher 1 (BHQ1) at the 3′ end.

We also synthesized the primers and probe that were described previously by McBride *et al*.^13^. These were renamed here as indicated in the parentheses below. The primer sequences were: forward primer (98 F), 5′-CATGGTGAAACCCCGTCTCTA-3′; reverse primer (188 R), 5′-GCCTCAGCCTCCCGAGTAG-3′. The sequence of the probe (133FH) was 5′-ATTAGCCGGGCGTGGTGGCG-3′, and it was labeled with 6-FAM at the 5′ end and TAMRA at the 3′ end.

### BLAST search

For homology searching, the BLAST program on the NCBI website was used (https://blast.ncbi.nlm.nih.gov/Blast.cgi)^48,49^. The following search databases were used: mouse, Genome (all assemblies top-level, Annotation Release 105) or Genome (GRCm38.p4 reference assembly top-level, Annotation Release 106); rat, Genome (all assemblies, Annotation Release 105); guinea pig, Genome (Cavpor3.0 reference Annotation Release 102); human (GRCh38.p7 reference assembly top-level, Annotation Release 108). Unless otherwise specified in the text, the parameters used were as follows: Program Selection, megablast; Short queries, off; Expect threshold, 10; Word size, 16; Match/Mismatch Scores, 1, −4; Gap Costs, 5, 2; Filters and Masking, all off.

### Primer3

Candidate primers and probes for the Alu model sequence were designed using Primer3web version 4.0.0 (http://bioinfo.ut.ee/primer3/)^40^. Parameters that differed from the default settings were as follows: Task, pick_primer_list; Mispriming Library (repeat library), RODENT_AND_SIMPLE. The Tm values of the primers and probes were calculated according to the default parameters of Primer3web.

### Prediction of primer and probe secondary structures

Unimolecular and bimolecular secondary structures of primers and probes were predicted by RNAstructure (version 5.6)^41^. Bimolecular structures of homo- and heterodimers were predicted using the “Fold DNA Bimolecular” function with forcing of the “Forbid Unimolecular Pairs” function. Only structures with the lowest free energy were considered.

### Genome extraction

The human and mouse genomes were extracted according to a standard method^25^, with slight modifications as described below.

To isolate human genomic DNA, NHDF cells in a 10-cm plate were trypsinized and collected by centrifugation at 400 × *g* for 5 min. After aspiration of the supernatant, the cells were resuspended in 10 ml of TBS buffer (137 mM NaCl, 5.4 mM KCl, 5 mM Tris-HCl, pH 7.4) and counted. Cells were aliquoted at 1 million cells per 1.5-ml tube and pelleted by centrifugation at 1,000 × *g* for 10 min at 4 °C. After the supernatant was removed by aspiration, the cells were resuspended in 50 μl of TE buffer (10 mM Tris-HCl, 1 mM EDTA, pH 8.0) per tube. Then, 450 μl of lysis buffer (10 mM Tris-HCl, 100 mM EDTA, 0.5% SDS, pH 8.0) containing 200 μg/ml RNase A (Nippon Gene, Chiyoda-ku, Japan) and 1,000 U/ml RNase T1 (Thermo Fisher Scientific) was added, and the lysate was incubated for 1 hour at 37 °C. After the incubation, 5 μl of 20 mg/ml Proteinase K solution was added to a final concentration of 100 μg/ml, and the lysate solution was incubated overnight at 50 °C. DNA was purified by phenol/chloroform/isoamyl alcohol extraction twice. Genomic DNA was precipitated with a 0.2 volume of 10 M ammonium acetate and a 2.5 volume of 100% ethanol, and rinsed with 70% ethanol. The precipitated DNA was dried at room temperature for 10 minutes, and dissolved in TE buffer.

Mouse genomic DNA was isolated from the lung, liver, and kidney. Fresh-frozen organs in liquid N_2_ were cut into small pieces and incubated in 3 ml of lysis buffer containing RNase A and T1 for 1 hour at 37 °C. After the addition of proteinase K, the solution was incubated overnight at 50 °C. After this step, the rodent genomic DNA was extracted as described for the extraction of human genomic DNA.

### Quantification of genomic DNA

Extracted genomic DNA was quantified using a NanoDrop UV spectrophotometer (Thermo Fisher Scientific) and a Quant-iT PicoGreen dsDNA Kit (Thermo Fisher Scientific). The PicoGreen assays were performed according to the manufacturer’s instructions, and fluorescence was measured using a microplate reader (Infinite M1000; TECAN, Männedorf, Switzerland). The amount of genomic DNA for Alu-qPCR was adjusted using the PicoGreen method.

### qPCR

Alu-qPCR with our primers (101 F and 206 R) and probe (144RH) was performed in a volume of 20 μl that contained 10 μl of TaqMan Universal Master Mix II, no UNG (Thermo Fisher Scientific), 0.2 μM forward and reverse primers, 0.25 μM hydrolysis probe, and an appropriate amount of genomic DNA, on an Applied Biosystems 7500 real-time PCR instrument (ABI 7500; Thermo Fisher Scientific). Easy Dilution Buffer (Takara, Kusatsu, Japan) was used to prepare serial dilutions of human genomic DNA only. TE buffer was used to prepare serial dilutions of human genomic DNA mixed with rodent genomic DNA, and each qPCR reaction was performed using a total of 100 ng of genomic DNA. The PCR conditions were 1 cycle of 95 °C for 10 min, followed by 50 cycles of 95 °C for 15 s, 56 °C for 30 s and 72 °C for 30 s.

Alu-qPCR with the primers (98 F and 188 R) and probe (133FH) of McBride *et al*. was performed essentially as described previously^13,44^. The qPCR was performed in a volume of 20 μl that contained 10 μl of TaqMan Universal Master Mix II, no UNG, 0.9 μM forward and reverse primers, 0.25 μM hydrolysis probe, and an appropriate amount of genomic DNA, on the ABI 7500. Samples were prepared as described above. The PCR conditions were 1 cycle of 50 °C for 2 min and 95 °C for 10 min, followed by 50 cycles of 95 °C for 15 s and 60 °C for 1 min.

The C_T_ values were calculated using SDS software (Thermo Fisher Scientific) with the default settings.

### Xenotransplantation subrenal capsule assay

Ten-fold serial dilutions of NHDF cells, consisting of 1,000 cells, 100 cells, and 10 cells, were prepared in triplicate (N = 3) in 50 μl of PBS on ice. After the backs of retired female C57BL/6 mice were surgically opened to expose the kidneys, the cell solutions were injected into the subrenal capsules of the kidneys, and then the implantation sites were sutured. Twelve hours after the procedure, the mice were euthanatized and the kidneys were surgically removed. The kidneys were snap-frozen in liquid nitrogen and stored at −80 °C before isolating the genomic DNA. Each whole kidney was subjected to genome extraction. qPCR calibration curves were prepared using a mixture of 10-fold serial dilutions of human genomic DNA with genomic DNA extracted from the kidneys of control non-injected mice (N = 3). The number of NHDF cells contained in the whole kidney was calculated from the amount of human genomic DNA per 100 ng of extracted DNA in the Alu-qPCR experiment and the total amount of extracted DNA.

### Tail vein injection of hMSCs

Aliquots of 0.5 million hMSCs at passage 5 in 100 μl of ice-cold PBS were intravenously injected into seven-week-old male NOD/SCID mice (N = 18) via a tail vein. As negative controls, organs from NOD/SCID mice (N = 3) that had not received injections of hMSCs were used. Whole lungs, livers, and kidneys were surgically removed at 1 (N = 6), 3 (N = 6), or 7 (N = 6) days after the injection. The organs were snap-frozen in liquid nitrogen and stored at −80 °C before isolating genomic DNA. Using the genomic DNA extracted from each organ, qPCR experiments were performed for triplicate samples in one run. The number of human cells in each organ was estimated as described in the *Xenotransplantation subrenal capsule assay* section.

### Data availability

All data values supporting the experimental conclusions are included in this article and its supplementary information. Source data are available from corresponding author.

## Electronic supplementary material

Supplementary Information
