# Supplementary material for: Highly sensitive and specific Alu-based quantification of human cells among rodent cells
Source: Sci Rep. 2017 Oct 16;7:13202. doi: 10.1038/s41598-017-13402-3 (PMC5643497; doi:10.1038/s41598-017-13402-3)
Supplement: Supplementary file 1 — Supplementary Information [file 41598_2017_13402_MOESM1_ESM.pdf]

Supplementary Information

Highly sensitive and specific Alu-based quantification of human cells among rodent cells

Kodai Funakoshi[1], Mozhdeh Bagheri[2], Ming Zhou[3], Ryoji Suzuki[3], Hiroshi Abe[3] & Hideo Akashi[3,*]

[1]Department of Microbiology and Immunology, Tohoku University Graduate School of Medicine, 2-1 Seiryo-machi, Aoba-ku, Sendai 980-8575, Japan

[2]Department of Molecular Pathology, Tohoku University Graduate School of Medicine, 2-1 Seiryo-machi, Aoba-ku, Sendai 980-8575, Japan

[3]Department of Anatomy, Akita University Graduate School of Medicine, 1-1-1 Hondo, Akita 010-8543, Japan

* To whom correspondence should be addressed. Tel: 81(Japan)-18-884-6055; Fax: 81(Japan)-18-884-6440; Email: akashi@gipc.akita-u.ac.jp

Table S1. Position weight matrix of the Alu consensus. The total numbers of A, G, T, and C at each position with non-redundant hits for the 46 Alu subfamily members in the Dfam 1.3 database are shown.

Position	1	2	3	4	5	6	7	8	9	10	11	12	13	14	15	16	17	18	19	20
Consensus	G	G	C	C	G	G	G	C	G	C	G	G	T	G	G	C	T	C	A	C
A	0	0	0	0	0	0	0	0	0	0	0	0	0	0	0	0	0	0	996,723	15,084
G	996,723	996,723	0	0	996,723	996,723	996,723	0	996,723	0	996,723	996,723	0	996,723	996,723	0	0	0	0	0
T	0	0	0	1,130	0	0	0	0	0	0	0	0	996,723	0	0	0	996,723	0	0	198,332
C	0	0	996,723	995,593	0	0	0	996,723	0	996,723	0	0	0	0	0	996,723	0	996,723	0	783,307

Position	21	22	23	24	25	26	27	28	29	30	31	32	33	34	35	36	37	38	39	40
Consensus	G	C	C	T	G	T	A	A	T	C	C	C	A	G	C	A	C	T	T	T
A	0	0	0	0	0	0	996,723	996,723	0	0	0	0	996,723	0	0	996,723	0	0	0	0
G	996,723	0	0	0	996,723	0	0	0	0	0	0	0	0	996,723	0	0	0	0	0	0
T	0	0	3,654	996,723	0	996,723	0	0	996,723	0	0	0	0	0	0	0	0	996,723	996,723	996,723
C	0	996,723	993,069	0	0	0	0	0	0	996,723	996,723	996,723	0	0	996,723	0	996,723	0	0	0

Position	41	42	43	44	45	46	47	48	49	50	51	52	53	54	55	56	57	58	59	60
Consensus	G	G	G	A	G	G	C	C	G	A	G	G	C	G	G	G	C	G	G	A
A	0	0	0	996,723	0	0	0	18,507	0	996,723	0	664	0	0	0	0	239,274	0	0	996,723
G	996,723	996,723	996,723	0	996,723	996,723	0	0	996,723	0	996,723	996,059	0	996,723	996,723	996,723	0	996,723	996,723	0
T	0	0	0	0	0	0	0	68,399	0	0	0	0	0	0	0	0	352,894	0	0	0
C	0	0	0	0	0	0	996,723	909,817	0	0	0	0	996,723	0	0	0	404,555	0	0	0

Position	61	62	63	64	65	66	67	68	69	70	71	72	73	74	75	76	77	78	79	80
Consensus	T	C	A	C	T	T	G	A	G	G	T	C	A	G	G	A	G	T	T	C
A	0	0	757,449	0	0	0	1,130	996,723	0	0	0	0	941,190	32,280	0	996,723	0	208,785	0	0
G	0	0	239,274	0	0	0	995,593	0	996,723	869,638	0	0	55,533	964,443	996,723	0	996,723	0	0	0
T	996,723	84,732	0	24,308	389,508	695,311	0	0	0	0	692,037	0	0	0	0	0	0	787,938	996,723	0
C	0	911,991	0	972,415	305,803	0	0	0	0	127,085	304,686	996,723	0	0	0	0	0	0	0	996,723

Position	81	82	83	84	85	86	87	88	89	90	91	92	93	94	95	96	97	98	99	100
Consensus	G	A	G	A	C	C	A	G	C	C	T	G	G	C	C	A	A	C	A	T
A	6,979	996,723	0	996,723	0	0	992,453	0	0	0	0	0	55,533	0	0	992,453	992,453	16,361	996,723	2,869
G	989,744	0	996,723	0	0	0	0	787,938	0	0	0	992,453	936,920	239,274	0	0	0	14,933	0	0
T	0	0	0	0	0	0	0	204,515	0	1,130	988,744	0	0	0	166,918	0	0	12,674	0	846,161
C	0	0	0	0	996,723	996,723	0	0	992,453	991,323	3,709	0	0	753,179	825,535	0	0	948,485	0	147,693

Position	101	102	103	104	105	106	107	108	109	110	111	112	113	114	115	116	117	118	119	120
Consensus	G	G	T	G	A	A	A	C	C	C	C	G	T	C	T	C	T	A	C	T
A	153,131	0	55,533	65,412	996,723	843,592	996,723	0	0	0	0	0	0	0	0	0	0	996,723	0	239,274
G	843,592	996,723	0	931,311	0	153,131	0	0	0	0	0	996,723	0	0	0	0	0	0	0	0
T	0	0	875,778	0	0	0	0	0	0	0	0	0	996,723	0	996,723	0	996,723	0	0	757,449
C	0	0	65,412	0	0	0	0	996,723	996,723	996,723	996,723	0	0	996,723	0	996,723	0	0	996,723	0

Position	121	122	123	124	125	126	127	128	129	130	131	132	133	-	134	135	136	137	138	139
Consensus	A	A	A	A	A	T	A	C	A	A	A	A	A	-	T	T	A	G	C	C
A	996,723	996,723	996,723	996,723	984,071	0	943,486	0	996,723	996,723	996,723	996,723	996,723	191,481	0	0	996,723	0	0	0
G	0	0	0	0	0	0	9,447	0	0	0	0	0	0	0	0	0	0	996,723	0	0
T	0	0	0	0	0	996,723	43,790	112,189	0	0	0	0	0	0	996,723	984,071	0	0	0	37,597
C	0	0	0	0	12,652	0	0	884,534	0	0	0	0	0	0	0	0	0	0	996,723	959,126

Position	140	141	142	143	144	145	146	147	148	149	150	151	152	153	154	155	156	157	158	159
Consensus	G	G	G	C	G	T	G	G	T	G	G	C	G	C	G	T	G	C	C	T
A	0	0	0	0	664	7,792	16,695	824	0	0	0	0	24,963	0	120,945	0	0	0	0	0
G	996,723	996,723	996,723	0	996,059	0	980,028	995,899	0	996,723	996,723	0	971,760	333,732	875,778	0	996,723	0	0	0
T	0	0	0	0	0	980,240	0	0	996,723	0	0	41,141	0	0	0	511,042	0	0	0	996,723
C	0	0	0	996,723	0	8,691	0	0	0	0	0	955,582	0	662,991	0	485,681	0	996,723	996,723	0

Position	160	161	162	163	164	165	166	167	168	169	170	171	172	173	174	175	176	177	-	178
Consensus	G	T	A	A	T	C	C	C	A	G	C	T	A	C	T	C	G	G	-	G
A	0	0	982,987	521,213	0	0	0	0	996,723	0	0	0	987,276	0	664	0	0	0	699	0
G	996,723	0	13,736	475,510	0	0	0	0	0	996,723	0	0	9,447	0	333	344	996,723	996,723	0	996,723
T	0	996,723	0	0	996,723	0	0	56,442	0	0	0	996,723	0	0	995,726	187,236	0	0	0	0
C	0	0	0	0	0	996,723	996,723	940,281	0	0	996,723	0	0	996,723	0	809,143	0	0	0	0

Position	179	180	181	182	183	184	185	186	187	188	189	190	191	192	193	194	195	196	197	198
Consensus	A	G	G	C	T	G	A	G	G	C	A	G	G	A	G	A	A	T	C	G
A	996,723	0	0	0	0	0	996,723	0	0	0	842,181	0	0	996,723	0	757,449	996,723	0	0	0
G	0	996,723	996,723	0	0	996,723	0	996,723	996,723	0	154,542	931,311	989,744	0	996,723	239,274	0	0	150,562	996,723
T	0	0	0	0	996,723	0	0	0	0	154,542	0	0	0	0	0	0	0	996,723	6,979	0
C	0	0	0	996,723	0	0	0	0	0	842,181	0	65,412	0	0	0	0	0	0	839,182	0

Position	199	200	201	202	203	204	205	206	207	-	208	209	210	211	212	213	214	215	216	217
Consensus	C	T	T	G	A	A	C	C	C	-	G	G	A	A	G	G	C	G	G	A
A	0	199	0	0	996,723	757,449	5,417	0	5,317	0	153,330	199	5,751	995,032	2,869	0	0	0	0	996,723
G	0	150,363	0	996,723	0	239,274	0	0	0	0	843,393	996,524	990,972	1,691	993,854	838,275	0	757,449	996,723	0
T	0	846,161	996,723	0	0	0	0	0	89,404	0	0	0	0	0	0	158,448	239,274	239,274	0	0
C	996,723	0	0	0	0	0	991,306	996,723	902,002	1,691	0	0	0	0	0	0	757,449	0	0	0

Position	218	219	220	221	222	223	224	225	226	227	228	229	230	231	232	233	234	235	236	237
Consensus	G	G	T	T	G	C	A	G	T	G	A	G	C	C	G	A	G	A	T	C
A	0	0	0	0	43,790	0	941,190	0	0	0	996,723	0	0	0	153,131	757,250	0	983,829	0	14,257
G	996,723	846,161	0	0	952,933	0	55,533	996,723	0	996,723	0	996,723	0	334	843,592	199	996,723	12,894	0	0
T	0	0	799,548	996,723	0	0	0	0	996,723	0	0	0	664	153,973	0	239,274	0	0	996,723	75,353
C	0	150,562	197,175	0	0	996,723	0	0	0	0	0	0	996,059	842,416	0	0	0	0	0	907,113

Position	238	239	240	241	242	243	244	245	246	247	248	249	250	251	252	-	-	-	-	-
Consensus	G	C	G	C	C	A	C	T	G	C	A	C	T	C	C	-	-	-	-	-
A	0	0	0	0	0	993,791	0	334	0	0	993,791	1,130	0	0	0	0	0	2,869	0	0
G	977,430	0	996,723	0	0	0	0	0	993,791	0	0	4,189	0	0	0	2,869	0	0	2,869	0
T	0	208,909	0	0	0	0	104,074	993,457	0	0	0	0	992,949	0	0	0	0	0	0	2,869
C	19,293	787,814	0	996,723	996,723	0	889,717	0	0	993,791	0	988,472	842	993,791	993,791	0	2,869	0	0	0

Position	-	-	253	254	255	256	257	258	259	260	261	262	263	264	265	-	266	267	268	269
Consensus	-	-	A	G	C	C	T	G	G	G	C	G	A	C	A	-	G	A	G	C
A	0	0	985,382	2,131	0	0	0	0	0	0	0	86,252	996,723	12,997	996,723	134,793	0	995,032	334	0
G	0	0	5,120	991,660	0	0	0	993,791	993,791	956,194	4,337	906,386	0	0	0	0	996,723	0	994,698	0
T	0	0	0	0	0	199	993,791	0	0	0	325,312	1,130	0	0	0	0	0	0	0	369,058
C	2,869	2,869	3,289	0	993,791	993,592	0	0	0	0	664,142	2,955	0	983,726	0	0	0	0	0	627,665

Position	270	271	272	273	274	275	276	277	278	279	280	281	282	283	284	285	286	287	288	289
Consensus	G	A	G	A	C	T	C	T	G	T	C	T	C	A	A	A	A	A	A	A
A	0	995,593	131,120	996,723	0	0	0	0	0	0	0	0	0	996,723	996,723	996,723	996,723	996,723	996,723	996,723
G	996,723	1,130	865,603	0	0	0	0	0	996,723	0	0	0	0	0	0	0	0	0	0	0
T	0	0	0	0	0	757,449	4,337	565,935	0	996,723	0	996,723	0	0	0	0	0	0	0	0
C	0	0	0	0	996,723	239,274	992,386	430,788	0	0	996,723	0	996,723	0	0	0	0	0	0	0

Position	290	291	292	293	294	295	296	297	298	299	300
Consensus	A	A	A	A	A	A	A	A	A	A	A
A	996,723	996,723	996,723	996,723	996,723	996,723	996,723	996,723	996,723	996,723	996,723
G	0	0	0	0	0	0	0	0	0	0	0
T	0	0	0	0	0	0	0	0	0	0	0
C	0	0	0	0	0	0	0	0	0	0	0

Table S2. Numbers of 19-nt 100%-matched sequences in the Alu model sequence with the mouse, rat, or guinea pig genome. The number of BLAST hits is indicated below the first position of each 19-nt contiguous sequence within the Alu model sequence. Sequences of 20 nt or longer starting from the position shown in red contained multiple 19-nt sequences that matched 100% with rodent genomes.

Position	1	2	3	4	5	6	7	8	9	10	11	12	13	14	15	16	17	18	19	20	21	22	23	24	25	26	27	28	29	30
Nucleotide	G	G	C	C	G	G	G	C	G	C	G	G	T	G	G	C	T	C	A	C	G	C	C	T	G	T	A	A	T	C
Mouse	0	0	0	0	0	2	2	2	2	4	30	26	31	28	34	43	32	240	242	270	931	83	113	119	16	45	32	31	29	2
Rat	0	0	0	0	0	0	0	0	0	0	24	19	27	32	40	42	40	226	187	172	616	81	92	94	15	37	32	27	25	0
Guinea pig	0	2	2	3	5	6	6	6	8	10	1,162	1,234	1,298	1,301	1,382	1,397	1,339	1,994	1,918	1,915	2,171	263	285	298	134	182	146	117	108	14

Position	31	32	33	34	35	36	37	38	39	40	41	42	43	44	45	46	47	48	49	50	51	52	53	54	55	56	57	58	59	60
Nucleotide	C	C	A	G	C	A	C	T	T	T	G	G	G	A	G	G	C	C	G	A	G	G	C	G	G	G	C	G	G	A
Mouse	0	0	0	0	0	0	0	2	0	0	0	0	0	0	0	0	0	0	0	0	0	0	0	0	0	0	0	2	0	0
Rat	0	0	2	2	2	0	0	0	2	0	0	0	0	0	0	0	0	0	0	0	0	0	0	0	0	0	0	1	1	2
Guinea pig	11	14	15	14	15	0	0	0	1	1	2	0	0	0	0	0	0	0	0	0	0	0	0	0	0	0	0	0	0	1

Position	61	62	63	64	65	66	67	68	69	70	71	72	73	74	75	76	77	78	79	80	81	82	83	84	85	86	87	88	89	90
Nucleotide	T	C	A	C	T	T	G	A	G	G	T	C	A	G	G	A	G	T	T	C	G	A	G	A	C	C	A	G	C	C
Mouse	2	2	0	0	0	0	0	0	4	0	6	6	13	38	2,709	43	33	9	7	7	7	4	0	3	0	0	0	0	0	0
Rat	2	2	0	0	0	0	0	0	2	2	8	13	15	34	412	28	18	0	0	0	2	0	0	2	0	0	2	0	0	0
Guinea pig	2	1	0	0	0	0	0	0	0	1	2	9	22	178	2,117	78	40	1	1	1	1	0	0	1	1	1	2	0	0	0

Position	91	92	93	94	95	96	97	98	99	100	101	102	103	104	105	106	107	108	109	110	111	112	113	114	115	116	117	118	119	120
Nucleotide	T	G	G	C	C	A	A	C	A	T	G	G	T	G	A	A	A	C	C	C	C	G	T	C	T	C	T	A	C	T
Mouse	0	0	0	0	0	0	2	2	0	0	0	0	0	0	0	0	0	0	0	0	0	0	2	0	0	0	2	0	0	2
Rat	0	0	0	0	0	0	2	4	0	0	0	0	0	0	0	0	0	0	0	0	0	0	0	6	6	0	4	4	0	0
Guinea pig	0	0	0	0	0	0	1	1	0	0	0	0	0	0	0	0	0	0	0	0	0	0	0	0	0	1	0	0	0	0

Position	121	122	123	124	125	126	127	128	129	130	131	132	133	134	135	136	137	138	139	140	141	142	143	144	145	146	147	148	149	150
Nucleotide	A	A	A	A	A	T	A	C	A	A	A	A	A	T	T	A	G	C	C	G	G	G	C	G	T	G	G	T	G	G
Mouse	0	0	0	0	0	0	0	0	6	11	17	26	43	137	431	18	1	0	0	0	2	2	2	2	2	2	4	2	2	2
Rat	0	0	0	0	0	0	0	0	0	0	0	0	0	0	0	0	0	0	0	0	0	0	0	0	4	2	2	2	2	6
Guinea pig	0	0	0	0	0	0	0	0	0	1	3	3	5	1	5	0	0	0	0	1	2	2	1	3	5	4	5	5	4	5

Position	151	152	153	154	155	156	157	158	159	160	161	162	163	164	165	166	167	168	169	170	171	172	173	174	175	176	177	178	179	180
Nucleotide	C	G	C	G	T	G	C	C	T	G	T	A	A	T	C	C	C	A	G	C	T	A	C	T	C	G	G	G	A	G
Mouse	4	22	10	7	19	67	51	2	2	2	4	4	6	12	8	8	8	14	12	12	40	153	162	141	131	347	629	732	411	24
Rat	6	29	6	6	25	67	56	4	2	2	2	4	7	11	9	12	12	13	10	16	45	179	192	178	167	356	613	740	408	23
Guinea pig	7	49	7	6	92	93	107	12	12	10	21	23	27	28	31	39	41	38	42	51	149	1,151	1,289	1,474	1,564	2,009	2,916	2,901	1,913	209

Position	181	182	183	184	185	186	187	188	189	190	191	192	193	194	195	196	197	198	199	200	201	202	203	204	205	206	207	208	209	210
Nucleotide	G	C	T	G	A	G	G	C	A	G	G	A	G	A	A	T	C	G	C	T	T	G	A	A	C	C	C	G	G	G
Mouse	17	9	4	4	4	2	0	0	0	0	0	0	0	0	0	0	0	0	0	0	0	0	0	0	0	0	0	0	0	0
Rat	10	6	3	0	0	0	0	0	0	0	0	0	0	0	0	0	0	0	0	0	0	0	0	0	0	0	0	0	0	0
Guinea pig	136	78	11	7	3	1	0	0	0	0	0	0	0	0	0	0	0	0	0	0	0	0	0	0	0	0	0	0	0	0

Position	211	212	213	214	215	216	217	218	219	220	221	222	223	224	225	226	227	228	229	230	231	232	233	234	235	236	237	238	239	240
Nucleotide	A	G	G	C	G	G	A	G	G	T	T	G	C	A	G	T	G	A	G	C	C	G	A	G	A	T	C	G	C	G
Mouse	0	0	0	0	0	0	0	0	0	0	0	0	0	0	0	0	0	0	0	0	0	0	0	0	0	0	0	0	0	2
Rat	0	0	0	0	0	0	0	0	0	0	0	0	0	0	0	0	0	0	0	0	0	0	0	0	0	0	0	0	0	0
Guinea pig	0	0	0	0	0	0	0	0	0	0	0	0	0	0	0	0	0	0	0	0	0	0	0	0	0	0	0	1	0	64

Position	241	242	243	244	245	246	247	248	249	250	251	252	253	254	255	256	257	258	259	260	261	262	263	264
Nucleotide	C	C	A	C	T	G	C	A	C	T	C	C	A	G	C	C	T	G	G	G	C	G	A	C
Mouse	2	2	0	0	0	0	0	0	0	0	6	0	0	0	0	0	0	0	0	0	0	0	2	2
Rat	0	0	0	0	0	0	0	0	0	0	0	0	0	0	0	0	0	0	0	0	0	2	4	4
Guinea pig	65	64	65	0	0	0	0	0	0	0	0	0	0	0	1	1	1	1	1	1	1	1	7	7

Table S3. Prediction of secondary structures of heterodimers for forward and reverse primers. The primer names, e.g., “54F” and “206R” mean a forward and a reverse primer whose 5′ ends start from position 54 and 206 in the Alu model sequence, respectively. “5-3t,−5.8” indicates 3 Ss in the 5-nt contiguous stem at the 3′-terminus with a free energy of −5.8 kcal/mol.

Length		20	20	21	20	21
	Name	206R	207R	207R	208R	208R
20	54F	5-2,−5.6	5-2,−5.6	5-2,−5.6	5-2,−5.6	5-2,−5.6
20	55F	5-2,−5.6	5-2,−5.6	5-2,−5.6	5-2,−5.6	5-2,−5.6
20	56F	5-2,−5.6	5-2,−5.6	5-2,−5.6	5-2,−5.6	5-2,−5.6
21	56F	5-3t,−5.8	5-3t,−5.7	5-3t,−5.8	5-2,−5.6	5-3t,−5.7
21	63F	6-4,−7.8	6-4t,−7.7	6-4,−7.8	5-3t,−5.8	6-4t,−7.7
20	64F	6-4,−7.8	6-4t,−7.7	6-4,−7.8	5-3t,−5.8	6-4t,−7.7
21	64F	6-4,−7.8	6-4t,−7.7	6-4,−7.8	5-3t,−5.8	6-4t,−7.7
20	101F	4-2,−4.1	5-3,−5.9	5-3,−5.9	5-3,−6.0	5-3,−6.0

Table S4. Features of Alu-qPCR primers and probes and MegaBLAST searches for rodent and human genomes. In column “0”, the numbers of 100%-matched hits of 20-nt subsequence (for a length of 20 nt or more) or of full-length sequence (for a length of 19 nt or less) are shown. In column “−1”, the number of 100%-matched hits of 19-nt (for a length of 20 nt or more) or of length−1-nt (for a length of 19 nt or less) subsequences are shown. In column “−2”, the number of 100%-matched hits of 18-nt (length of 20 nt or more) or of length−2-nt (for a length of 19 nt or less) subsequences are shown. The following search databases were used: mouse, Genome (all assemblies top-level, Annotation Release 106); rat, Genome (all assemblies, Annotation Release 106); guinea pig, Genome (Cavpor3.0 reference Annotation Release 102); human, Genome (all assemblies top-level, Annotation Release 108). * Tm values obtained without TaqMan-MGB effects. ** Hits include sequences within position 807-1566 in NW_007906637.

				Mouse			Rat			Guinea pig			Human	
Primer or probe sequence	Type	Length	Tm	0	−1	−2	0	−1	−2	0	−1	−2	0	Ref
CATGGTGAAACCCCGTCTCTA	F	21	59.5	0	1	0	0	4	4	0	1	0	60,664	1
GCCTCAGCCTCCCGAGTAG	R	19	61.5	7	20	197	23	41	310	91	316	3,737	112,211	1
ATTAGCCGGGCGTGGTGGCG	H	20	68.9	64	283	862	0	0	2	1	36	91	14,201	1
GACCATCCCGGCTAAAACG	F	19	58.6	0	0	0	0	0	0	0	0	0	5,116	2
CGGGTTCACGCCATTCTC	R	18	58.5	0	0	0	0	2**	2	0	0	0	31,656	2
CCCCGTCTCTACTAAA	H	16	48.5*	0	0	2	1**	2	15	0	1	1	107,029	2
GTCAGGAGATCGAGACCATCCT	F	22	60.8	0	0	0	2	0	0	0	0	0	60,769	3
AGTGGCGCAATCTCGGC	R	17	60.5	0	0	0	0	0	2	0	0	0	13,290	3
AGCTACTCGGGAGGCTGAGGCAGGA	H	25	71.4	176	48	46	248	91	108	3,916	661	946	132,529	3
CTTGCAGTGAGCCGAGATT	F	19	57.9	0	0	0	0	0	4	0	0	0	15,552	4
GAGACGGAGTCTCGCTCTGTC	R	21	61.9	0	0	0	0	0	0	0	1	0	28,269	4
ACTGCAGTCCGCAGTCCGGCCT	H	22	71.6*	0	0	0	0	0	0	0	0	0	3,916	4
GAGGCGGGCGGATCA	F	15	58.2	3	24	91	2	46	0	4	7	4	27,211	5
CCCGGCTAATTTTTGTATTTTTAGTAG	R	27	57.5	0	2	2	4	0	19**	1	2	16	192,061	5
CAGCCTGGCCAACATGGTGAAACC	H	24	67.6	0	0	3	0	4	2	1	1	0	143,855	5
GGTGAAACCCCGTCTCTACT	F	20	58.7	0	0	0	0	1**	0	0	0	0	89,063	This study
GGTTCAAGCGATTCTCCTGC	R	20	59.3	0	0	1	0	0	0	0	0	1	72,640	This study
CGCCCGGCTAATTTTTGTAT	H	20	57.8	0	0	1	0	0	0	0	0	1	9,838	This study

1. McBride, C., Gaupp, D. & Phinney, D. G. Quantifying levels of transplanted murine and human mesenchymal stem cells *in vivo* by real-time PCR. *Cytotherapy* **5**, 7-18 (2003).
2. Nicklas, J. A. & Buel, E. Simultaneous determination of total human and male DNA using a duplex real-time PCR assay. *J. Forensic Sci.* **51**, 1005-1015 (2006).
3. Preston Campbell, J. *et al.* TRIzol and Alu qPCR-based quantification of metastatic seeding within the skeleton. *Sci. Rep.* **5**, 12635; 10.1038/srep12635 (2015).
4. Walker, J. A. *et al.* Multiplex polymerase chain reaction for simultaneous quantitation of human nuclear, mitochondrial, and male Y-chromosome DNA: application in human identification. *Anal. Biochem.* **337**, 89-97 (2005).
5. Zhang, W. *et al.* Development and qualification of a high sensitivity, high throughput Q-PCR assay for quantitation of residual host cell DNA in purification process intermediate and drug substance samples. *J. Pharm. Biomed. Anal.* **100**, 145-149 (2014).

Table S5. Applicability of our criteria to Alu-qPCR primers and MegaBLAST searches for rodent and human genomes. In column “0”, the numbers of 100%-matched hits of 20-nt subsequence (for a length of 20 nt or more) or full-length sequence (for a length of 19 nt or less) are shown. In column “−1”, the number of 100%-matched hits of 19 nt (for a length of 20 nt or more) or length−1-nt (for a length of 19 nt or less) subsequences are shown. In column “−2”, the number of 100%-matched hits of 18-nt (for a length of 20 nt or more) or of length−2-nt (for a length of 19 nt or less) subsequences are shown. The following search databases were used: mouse, Genome (all assemblies top-level, Annotation Release 106); rat, Genome (all assemblies, Annotation Release 106); guinea pig, Genome (Cavpor3.0 reference Annotation Release 102); human, Genome (all assemblies top-level, Annotation Release 108).

				Criteria							Mouse			Rat			Guinea pig			Human	
Primer sequence	Type	Length	Tm	i	ii	iii	iv	v	vi	vii	0	−1	−2	0	−1	−2	0	−1	−2	0	Ref
ACGCCTGTAATCCCAGCACTT	F	21	62.1		X		X	X		X	381	690	1,177	181	693	1,715	749	743	2,375	141,560	1, 5
TCGCCCAGGCTGGAGTGCA	R	19	66.4	X			X			X	0	0	5	0	0	21	0	0	85	80,747	1, 5
TGGCTCACGCCTGTAATCCCA	F	21	63.9		X		X		X	X	16	7	12	56	25	36	307	24	32	148,826	2
GCCACTACGCCCGGCTAATTT	R	21	63.4		X		X	X	X		1	4	4	0	0	0	1	2	3	8,449	2
GTCAGGAGATCGAGACCATCCC	F	22	61.7		X	X		X		X	0	0	0	2	0	0	0	0	0	117,628	3, 4
TCCTGCCTCAGCCTCCCAAG	R	20	63.8		X	X	X	X		X	491	560	483	910	760	930	565	200	755	85,430	3, 4
GTCAGGAGATCGAGACCATCCC	F	22	61.7		X	X		X	X	X	0	0	0	2	0	0	0	0	0	117,628	3, 4
CCACTACGCCCGGCTAATTT	R	20	60.5		X		X	X	X		1	0	5	0	0	0	0	1	3	8,414	4
GTCAGGAGATCGAGACCATCCC	F	22	61.7		X	X		X	X	X	0	0	0	2	0	0	0	0	0	117,628	3, 4
GCTCTGTCGCCCAGGCTGGAGT	R	22	68.7		X	X	X	X	X	X	0	3	2	0	0	0	0	0	6	74,429	4
CACCTGTAATCCCAGCACTTT	F	21	58.2		X		X			X	350	526	1,387	469	968	1,426	1,960	4,445	5,878	85,879	5
CCCAGGCTGGAGTGCAGT	R	18	61.6			X	X			X	4	0	9	12	6	17	76	7	18	273,278	5
CCTGAGGTCAGGAGTTCGAG	F	20	59.2		X	X	X	X		X	0	0	0	21	0	0	0	0	0	38,322	6
CCCGAGTAGCTGGGATTACA	R	20	58.6		X	X	X	X	X	X	2	0	3	4	0	6	24	14	27	51,754	6
GTGGCTCACGCCTGTAATC	F	19	58.6			X	X	X		X	18	14	18	35	62	37	433	44	25	157,768	6
CAGGCTGGAGTGCAGTGG	R	18	60.4			X	X	X		X	4	1	6	14	6	9	78	4	16	297,664	6
CGAGGCGGGTGGATCATGAGGT	F	22	66.4	X	X	X	X	X			0	0	0	0	0	2	0	1	0	11,079	7
TCTGTCGCCCAGGCCGGACT	R	20	68.0	X	X		X	X	X		0	0	0	0	0	0	0	0	0	3,845	7
GAGATCGAGACCACGGTGAAA	F	21	59.8	X	X		X			X	0	0	0	0	0	0	0	0	0	1,960	7
TTTGAGACGGAGTCTCGTT	R	19	56.0				X			X	0	0	1	2	1	0	3	1	38	1,806	7
CAACATAGTGAAACCCCGTCTCT	F	23	60.3		X	X	X	X	X	X	3	0	2	8	2	4	5	20	2	8,632	8
GCCTCAGCCTCCCGAGTAG	R	19	61.5			X	X	X	X	X	7	20	197	23	41	310	91	316	3,737	112,211	8

1. Mira, E., Lacalle, R. A., Gomez-Mouton, C., Leonardo, E. & Manes, S. Quantitative determination of tumor cell intravasation in a real-time polymerase chain reaction-based assay. *Clin. Exp. Metastasis* **19**, 313-318 (2002).
2. Nehmann, N., Wicklein, D., Schumacher, U. & Muller, R. Comparison of two techniques for the screening of human tumor cells in mouse blood: quantitative real-time polymerase chain reaction (qRT-PCR) versus laser scanning cytometry (LSC). *Acta Histochem.* **112**, 489-496 (2010).
3. Nicklas, J. A. & Buel, E. Development of an Alu-based, real-time PCR method for quantitation of human DNA in forensic samples. *J. Forensic Sci.* **48**, 936-944 (2003).
4. Opel, K. L., Fleishaker, E. L., Nicklas, J. A., Buel, E. & McCord, B. R. Evaluation and quantification of nuclear DNA from human telogen hairs. *J. Forensic Sci.* **53**, 853-857 (2008).
5. Schneider, T., Osl, F., Friess, T., Stockinger, H. & Scheuer, W. V. Quantification of human Alu sequences by real-time PCR--an improved method to measure therapeutic efficacy of anti-metastatic drugs in human xenotransplants. *Clin. Exp. Metastasis* **19**, 571-582 (2002).
6. Umetani, N. *et al.* Increased integrity of free circulating DNA in sera of patients with colorectal or periampullary cancer: direct quantitative PCR for ALU repeats. *Clin. Chem.* **52**, 1062-1069 (2006).
7. Walker, J. A. *et al.* Human DNA quantitation using Alu element-based polymerase chain reaction. *Anal. Biochem.* **315**, 122-128 (2003).
8. Witt, N. *et al.* An assessment of air as a source of DNA contamination encountered when performing PCR. *J. Biomol. Tech.* **20**, 236-240 (2009).

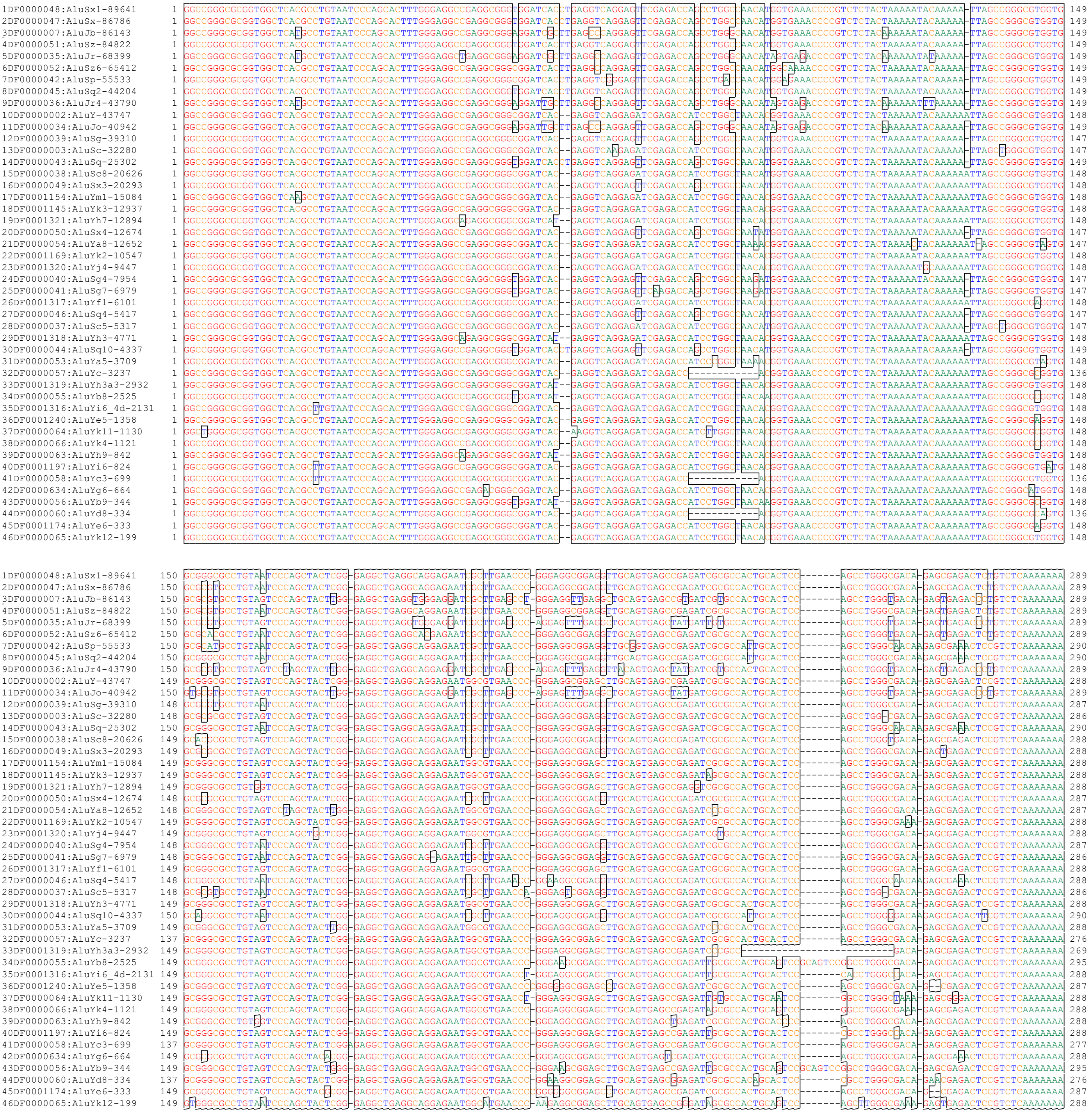

Figure S1. Alignment of the 46 Alu model-derived consensus sequences in Dfam1.3 using the ClustalW2 program. The order of alignment is followed by the number of non-redundant hits in the Dfam database. The alignment number, Dfam accession number, Alu subfamily name, and number of non-redundant hits are shown at left.

```
This study        1 GGCCGGGCGCGGTGGCTCACGCCTGTAATCCCAGCACTTTGGGAGGCCGAGGCGGGCGGA 60
AluSz(DF0000051)  1 GGCCGGGCGCGGTGGCTCACGCCTGTAATCCCAGCACTTTGGGAGGCCGAGGCGGGTGGA 60
AluSx(DF0000047)  1 GGCCGGGCGCGGTGGCTCACGCCTGTAATCCCAGCACTTTGGGAGGCCGAGGCGGGTGGA 60
Price             1 GGCCGGGCGCGGTGGCTCACGCCTGTAATCCCAGCACTTTGGGAGGCCGAGGCGGGCGGA 60
Kariya            1 GGCTGGGCGTGGTGGCTCACGCCTGTAATCCCAGCACTTTGGGAGGCCGAGGTGGGTGGA 60
Deininger         1 -GCTGGGCGTGGTGGCTCACACCTGTAATCCCAGCACTTTGGGAGGCCGAGGTGGGTGGA 59

This study       61 TCACTTGAGGTCAGGAGTTCGAGACCAGCCTGGCCAACATGGTGAAACCCCGTCTCTACT 120
AluSz(DF0000051) 61 TCACTTGAGGTCAGGAGTTCGAGACCAGCCTGGCCAACATGGTGAAACCCCGTCTCTACT 120
AluSx(DF0000047) 61 TCACCTGAGGTCAGGAGTTCGAGACCAGCCTGGCCAACATGGTGAAACCCCGTCTCTACT 120
Price            61 TCACCTGAGGTCAGGAGTTCGAGACCAGCCTGGCCAACATGGTGAAACCCCGTCTCTACT 120
Kariya           61 TCACCTGAGGTCAGGAGTTCAAGACCAGCCTGGCCAACATGGTGAAACCCCGTCTCTACT 120
Deininger        60 TCACCTGAGGTCAGGAGTTCAAGACCAGCCTGGCCAACATGGTGAAACCCCGTCTCTACT 119

This study       121 AAAAATACAAAAATTAGCCGGGCGTGGTGGCGCGTGCCTGTAATCCCAGCTACTCGGGAG 180
AluSz(DF0000051) 121 AAAAATACAAAAATTAGCCGGGCGTGGTGGCGCGTGCCTGTAATCCCAGCTACTCGGGAG 180
AluSx(DF0000047) 121 AAAAATACAAAAATTAGCCGGGCGTGGTGGCGCGTGCCTGTAATCCCAGCTACTCGGGAG 180
Price            121 AAAAATACAAAAATTAGCCGGGCGTGGTGGCGCGCGCCTGTAATCCCAGCTACTCGGGAG 180
Kariya           121 AAAAATACAAAAATTAGCCGGGCGTGGTGGCGCGCGCCTGTAATCCCAGCTACTCGGGAG 180
Deininger        120 AAAAATACAAAAATTAGCCGGGCGTGGTGGCGCGCGCCTGTAATCCCAGCTACTCGGGAG 179

This study       181 GCTGAGGCAGGAGAATCGCTTGAACCCGGGAGGCGGAGGTTGCAGTGAGCCGAGATCGCG 240
AluSz(DF0000051) 181 GCTGAGGCAGGAGAATCGCTTGAACCCGGGAGGCGGAGGTTGCAGTGAGCCGAGATCGCG 240
AluSx(DF0000047) 181 GCTGAGGCAGGAGAATCGCTTGAACCCGGGAGGCGGAGGTTGCAGTGAGCCGAGATCGCG 240
Price            181 GCTGAGGCAGGAGAATCGCTTGAACCCGGGAGGCGGAGGTTGCAGTGAGCCGAGATCGCG 240
Kariya           181 GCTGAGGCAGGAGAATCGCTTGAACCCGGGAGGTGGAGGTTGCAGTGAGCCGAGATCGCG 240
Deininger        180 GCTGAGGCAGGAGAATCGCTTGAACCCAGGAGGTGGAGGTTGCAGTGAGCCGAGATCGCG 239

This study       241 CCACTGCACTCCAGCCTGGGCGACAGAGCGAGACTCTGTCTC 282
AluSz(DF0000051) 241 CCACTGCACTCCAGCCTGGGCGACAGAGTGAGACTCTGTCTC 282
AluSx(DF0000047) 241 CCACTGCACTCCAGCCTGGGCGACAGAGCGAGACTCCGTCTC 282
Price            241 CCACTGCACTCCAGCCTGGGCGACAGAGCGAGACTCCGTCTC 282
Kariya           241 CCACTGCACTCCAGCCTGGGCGACAGAGCGAGACTCCGTCTC 282
Deininger        240 CCACTGCACTCCAGCCTGGGCAACAGAGCGAGACTCCATCTC 281
```

Figure S2. Alignment of the Alu consensus sequence. The Alu model sequences of AluSx, AluSz, Price et al., Kariya et al., Deininger et al., and this study were aligned by the ClustalW2 program. The Dfam accession numbers of AluSx and AluSz are also shown.

```
  1 GGCCGGGCGCGGTGGCTCACGCCTGTAATCCCAGCACTTTGGGAGGCCGA 50
    |||||||||||||||| ||||||||||:|||||||  :|:|||||||:||
  2 GGCCGGGCGCGGTGGCGCACGCCTGTAGTCCCAGCTACTCGGGAGGCTGA

 51 GGCGGGCGGATCACTTGAGGTCAGGAGTTCGAGACCAGCCTGGCCAACAT 100
    |||:|| |||||||||||| :||||||||||||:||||||||| ||||||
 51 GGCAGGAGGATCACTTGAGTCCAGGAGTTCGAGGCCAGCCTGGGCAACAT

101 GGTGAAACCCCGTCTCTACTAAAAATACAAAAATTAGCCGGGCGTGGTGG 150
    :|::|:||||:|||||                   |||||||||:|||||
101 AGCAAGACCCTGTCTC 116             1 AGCCGGGCGCGGTGG 15

151 CGCGTGCCTGTAATCCCAGCTACTCGGGAGGCTGAGGCAGGAGAATCGCT 200
    |||::|||||||:||||||||||||||||||||||||||||||:|||:||
 16 CGCACGCCTGTAGTCCCAGCTACTCGGGAGGCTGAGGCAGGAGGATCACT 65

201 TGAACCCGGGAGGCGGAGGTTGCAGTGAGCCGAGATCGCGCCACTGCACT 250
    |||::||:|||| : ||||----------------------------|||
 66 TGAGTCCAGGAGTTCGAGG----------------------------ACT 87

251 CCAGCCTGGGCGACAGAGCGAGACTCTGTCTCAAAAAAAAAAAAAAAAAA 300
    |||||||||||:||| |||:||||:||||||||||| ||||
 88 CCAGCCTGGGCAACATAGCAAGACCCTGTCTCAAAACAAAA 125
```

Figure S3. Local alignments of the model human Alu sequence (upper) with the mouse PB1 sequence (lower) using the Censor program. The mouse PB1 sequence was aligned with the model Alu sequence from positions 1 to 115 and from 136 to 291. The non-aligned sequence from positions 117 to 135 contained the Alu-specific A-rich sequence.

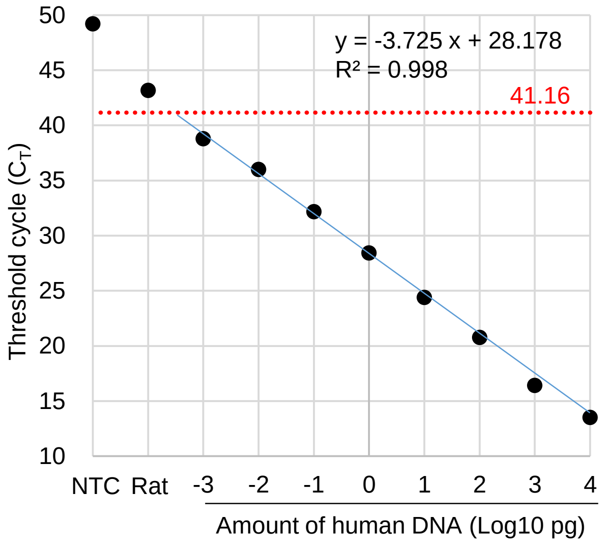

Figure S4. Standard curve for detecting human genomic DNA among a total of 100 ng of mixed genomic DNA from human and rat. qPCR was performed for duplicate samples in one run, and the mean C_T value was plotted. For non-detected samples, the C_T values were assumed to be 50 cycles. Negative control samples included TE buffer for the no-template control (NTC) and a sample of rat genomic DNA only (Rat). The threshold cycle of detection was set at 2 cycles below the C_T value for the sample of rodent genomic DNA only (red dotted line), and the number of cycles is shown in red. The equation for linear approximation (blue line) and the R^2 value are also shown.
